# Predicting PD-L1 in Lung Adenocarcinoma Using ^18^F-FDG PET/CT Radiomic Features

**DOI:** 10.3390/diagnostics15050543

**Published:** 2025-02-24

**Authors:** Huiyuan Zhang, Xiangxi Meng, Zhe Wang, Xin Zhou, Yang Liu, Nan Li

**Affiliations:** 1 Department of Nuclear Medicine, Beijing Chest Hospital, Capital Medical University/Beijing Tuberculosis and Thoracic Tumor Research Institute, Beijing 101149, China; 19801261125@163.com; 2 Key Laboratory of Carcinogenesis and Translational Research (Ministry of Education), Beijing Key Laboratory of Research, Investigation and Evaluation of Radiopharmaceuticals, NMPA Key Laboratory for Research and Evaluation of Radiopharmaceuticals (National Medical Products Administration), Department of Nuclear Medicine, Peking University Cancer Hospital & Institute, No. 52 Fucheng Rd, Beijing 100142, Chinaliuyang72021@163.com (Y.L.); 3 United Imaging Healthcare Group, Central Research Institute, Shanghai 201900, China

**Keywords:** PD-L1, [^18^F] FDG, PET/CT, radiomics, lung adenocarcinoma

## Abstract

**Background/Objectives**: This study aims to retrospectively analyze the clinical and imaging data of 101 patients with lung adenocarcinoma who underwent [^18^F]FDG PET/CT examination and were pathologically confirmed in the Department of Nuclear Medicine at Peking University Cancer Hospital. This study explores the predictive value and important features of [^18^F]FDG PET/CT radiomics for PD-L1 expression levels in lung adenocarcinoma patients, assisting in screening patients who may benefit from immunotherapy. **Methods**: 101 patients with histologically confirmed lung adenocarcinoma who received pre-treatment [^18^F] FDG PET/CT were included. Among them, 44 patients were determined to be PD-L1 positive and 57 patients were determined to be PD-L1 negative based on immunohistochemical assays. Clinical data, PET/CT radiomics parameters, conventional metabolic parameters, and observed CT characteristics were included in the modeling. Random Forest was used in feature denoising, while Forward Stepwise Regression and the Least Absolute Shrinkage and Selection Operator were used in feature selection. Models based on Tree, Discriminant, Logistic Regression, and Support Vector Machine were trained and evaluatedto explore the value of clinical data, PET/CT radiomics parameters, conventional metabolic parameters, and observed CT characteristics. **Results**: All models showed some predictive ability in distinguishing PD-L1 positive from PD-L1 negative samples. Among the multimodal imaging, clinical data were incorporated into the models, with clinical stage and gender selected by Forward Stepwise Regression, while clinical stage, smoking history, and gender were selected by LASSO. When incorporating clinical data and thin-section CT-derived images into the models, nodular type, spiculation, and CT Shape Flatness were selected by Forward Stepwise Regression, while nodular type and spiculation were selected by LASSO. When incorporating clinical data, PET/CT radiomics, observed CT characteristics, and conventional metabolic information. Forward Stepwise Regression selected TLGlean, MTV, nodule component, PET Shape Sphericity, while LASSO selected SULmax, MTV, nodular type, PET Shape Sphericity, and spiculation. **Conclusions:** The integration of clinical data, PET/CT radiomics, and conventional metabolic parameters effectively predicted PD-L1 expression, thereby assisting the selection of patients who would benefit from immunotherapy. Observed CT characteristics and conventional metabolic information play an important role in predicting PD-L1 expression levels.

## 1. Introduction

Lung cancer is the leading causeof nearly one-quarter of all cancer-related deaths worldwide [1]. Over 80% of lung malignancies belong to non-small cell lung cancer (NSCLC) [2], with adenocarcinoma being the major histologic subtype. Despite the availability of various therapies for NSCLC, including surgery, radiotherapy, and chemotherapy, the 5-year relative survival rate is still very low, at approximately 19% [1]. The pressing clinical demand for improved treatment modalities in non-small cell lung cancer (NSCLC) has been partially addressed by recent advances in immune checkpoint blockade therapy. In particular, the development of monoclonal antibodies targeting the programmed cell death protein 1 (PD-1) and the programmed death-ligand 1 (PD-L1) has demonstrated significant therapeutic efficacy, substantially improving progression-free survival and overall response rates in a subset of NSCLC patients [3]. PD-1 is expressed on the T cells’ surface and regulates their activation [4]. In tumor cells, PD-L1 overexpression serves as an immune evasion mechanism by binding to PD-1 receptors on T lymphocytes, thereby suppressing T cell activation and cytotoxic function, ultimately allowing tumor cells to escape immune surveillance [5]. Thus, tumor cells can establish a microenvironment conducive to sustained proliferation [6]. Several randomized studies with PD-1 and PD-L1 inhibitors have shown a greater survival benefit than traditional therapy in NSCLS patients [7,8,9,10,11]. Therefore, it is imperative to evaluate the expression of PD-1 and PD-L1 accurately.

Now, the identification of PD-L1 expression is mainly based on the immunohistochemistry of tumor specimens obtained by biopsy, which may not represent the whole tumor. PD-L1 expression in NSCLC has showed significant heterogeneity [12,13], which could lead to inaccurate results, especially when the testing is performed on a small tissue specimen [14]. Additionally, a biopsy as an invasive method is associated with high risks such as bleeding, pneumothorax, and tumor cell dissemination. Therefore, we urgently need a noninvasive method to predict PD-L1 expression using clinical information.

Noninvasive ^18^F-fluorodeoxyglucose positron emission tomography/computed tomography (^18^F-FDG PET/CT) can provide morphological and functional analysis simultaneously. It can also evaluate a tumor in its entirety, overcome the heterogeneity of tumor tissue, and be repeated easily at any point during treatment [15]. ^18^F-FDG PET/CT has been applied to diagnosis, staging, prognosis, and treatment response monitoring in NSCLC patients [16,17]. In recent years, numerous studies have investigated the relationship between ^18^F-FDG PET/CT parameters and PD-L1 expression in patients with non-small cell lung cancer (NSCLC). Findings suggest that metrics such as SUVmax and TLG may be associated with PD-L1 expression levels [18,19]. Several studies have also shown that radiomic signatures derived from CT imaging hold promise for predicting PD-L1 expression in NSCLC [20,21]. Radiomics is defined as the use of computer software to convert medical images to quantitative data and the application of statistical and/or machine learning methods to analyze these data, screening out the most valuable radiomic features to assist clinical decision-making [22]. As an emerging research methodology, radiomics has established a relatively comprehensive theoretical framework and standardized research pipeline. By overcoming the inherent limitations and subjectivity associated with conventional physician-based image interpretation, radiomics technology transforms imaging features into objective, quantifiable data for systematic analysis. This paradigm shift substantially enhances the clinical utility of medical imaging and holds significant implications for the burgeoning field of precision medicine.

To the best of our knowledge, few predictive models based on ^18^F-FDG PET/CT radiomics have been used to identify PD-L1 expression so far. Additionally, previous studies did not rigorously compare whether PET imaging provided predictive value beyond traditional clinical variables and anatomical CT features. This staged approach will establish whether metabolic imaging radiomics provides complementary biological information beyond conventional parameters, ultimately providing the best model choice for clinical implementation. Herein, the purpose of this study was to assess the radiomic features of ^18^F-FDG PET/CT in lung adenocarcinoma patients to identify PD-L1 expression noninvasively and, at the same time, to study the importance of different modal image radiomic features in prediction models.

## 2. Materials and Methods

**Figure 1:**
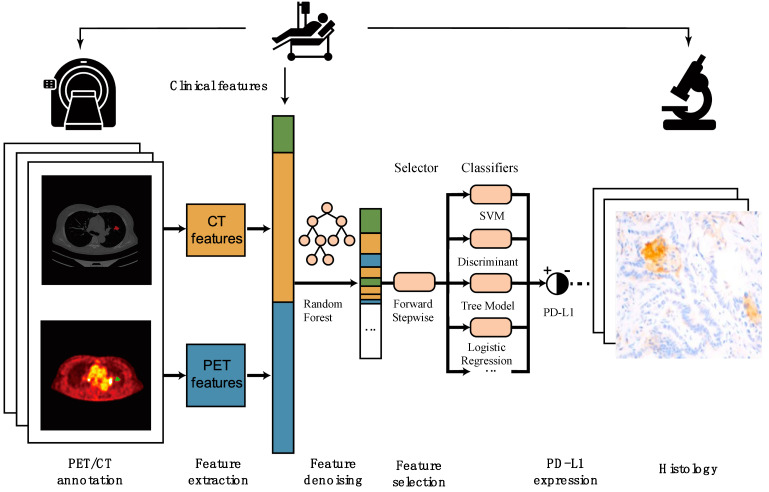
The workflow of our study.

The workflow is as follow (Figure 1):

### 2.1. Patients

We retrospectively collected data from patients who were clinicopathologically confirmed to have lung adenocarcinoma at Peking University Cancer Hospital. A total of 101 patients were included in this study, and they were randomly divided into the training cohort (*n* = 71) and the validation cohort (*n* = 30).

The inclusion criteria were as follows: (I) Pathologically proved lung adenocarcinoma; (II) IHC examination of PD-L1 performed; (III) Standard ^18^F-FDG PET/CT test performed before treatment; (IV) Complete clinical characteristics, including age, sex, smoking history, tumor size, and TNM staging. Exclusion criteria: (I) Therapy performed before^18^F-FDG PET/CT and IHC; (II) ^18^F-FDG PET/CT images with artifacts.

### 2.2. PD-L1 Detection

Immunohistochemistry was performed on 101 biopsy or surgery specimens using formalin-fixed tissue sections. Sections (4 μm thickness) were cut and stained with antibody clone 22C3. Tumor cells showing membranous staining for PD-L1 were evaluated as positive cells. The positive expression rate refers to the percentage of PD-L1-positive cells among all tumor cells in the entire section. A value of <1% was defined as negative expression, while ≥1% was considered PD-L1 positive expression. The results were independently assessed by two experienced pathologists. We retrospectively obtained PD-L1 expression data from our hospital’s medical record system.

### 2.3. PET/CT Imaging Protocol

For image analysis, pre-treatment PET/CT scans were used to obtain CT and PET texture analysis and FDG uptake. All patients fasted for at least 6 hours, and serum glucose concentration was confirmed to be less than 120 mg/dL before injecting ^18^F- FDG (0.1 mCi/Kg). Scans were performed from the top of the skull to the middle of the thigh 1 hour after ^18^F- FDG injection using a Philips Gemini TF 16 PET/CT (hilips Healthcare, Cleveland, OH, USA). PET imaging was performed in three-dimensional acquisition mode utilizing Flow Motion technology, with a continuous bed speed of 1.5 mm/s from the vertex to the upper thigh. The acquired PET data were reconstructed using an ordered-subsets expectation maximization (OSEM) algorithm (200 × 200 matrix, 2 iterations, and 11 subsets) following attenuation correction with low-dose CT images. A post-reconstruction Gaussian filter (5-mm kernel size) was applied to the PET images. For anatomical correlation and attenuation correction, high-resolution breath-hold CT scans were acquired using the following parameters: 120 kV tube voltage and 146 mAs tube current. CT images were reconstructed using the Sinogram Affirmed Iterative Reconstruction (SAFIRE) algorithm (512 × 512 matrix, and a slice thickness: 3 –5 mm.

### 2.4. Manual Segmentation

SUV-derived parameter acquisition: The region of interest (ROI) was set at the location of primary pulmonary lesion, and SUL-derived parameters, including MTV, SULmax, SULmin, SULmean, and TLGlean, were calculated using a Philips workstation. TLG was defined as SUVmean × MTV. The ROI on PET images was manually delineated by one radiologist and reviewed by another senior radiologist.

HRCT signs analyzed included: the long axis, short axis, and mean axis (half of the long axis and short axis) of the primary lesions, spicule sign, pleural indentation sign, and nodule component.

Segmentation of HRCT and PET images: Segmentation was performed with manual adjustment based on a 41% SUVmax threshold, using ITK-Snap software (Version 3.6, USA). Each primary lesion was manually contoured on HRCT and PET images by a radiologist and reviewed by another senior radiologist. The entire tumor, including necrotic or cystic areas, was segmented.

### 2.5. Radiomic Features and Model Building

Three categories of radiomic features were systematically extracted from the region of interest (ROI) using PyRadiomics (v3.0.1) in Python 3.6.2: (1) First-order statistics: Quantifying intensity distributions (e.g., energy, entropy, kurtosis). (2) Morphological features: Characterizing 3D shape and size properties (e.g., sphericity, surface area-to-volume ratio). (3) Texture features: Capturing spatial heterogeneity through gray-level co-occurrence matrix (GLCM), gray-level run-length matrix (GLRLM), and neighborhood gray-tone difference matrix (NGTDM).

To mitigate dimensionality and redundancy, a two-stage feature selection framework was implemented: Stage 1 (Denoising): A random forest-based filter method ranked features by Gini importance, retaining the top 30% with non-zero variance. Stage 2 (Refinement): Forward Stepwise Regression (AIC minimization) and LASSO regularization (λ selected via 10-fold cross-validation) were applied to identify optimal feature subsets. The LASSO and Forward Stepwise Regression methods were used because their performance has been validated in numerous previous studies as suitable for this specific task.

Four machine learning classifiers were trained and compared: Decision Tree (Gini impurity criterion, max depth = 5). Linear Discriminant Analysis (singular value decomposition solver). L2-penalized Logistic Regression (C = 1.0). Support Vector Machine (RBF kernel, γ = 0.1). Model performance was rigorously evaluated on the training cohort using 5-fold cross-validation, as well as the validation group, with accuracy and area under the ROC curve (AUC) as primary metrics.

### 2.6. Statistical Analysis

Model performance was assessed using accuracy, receiver operating characteristic curve (ROC), and the area under curve (AUC).

## 3. Results

### 3.1. Clinicopathological Characteristics:

A total of 101 patients (34–76 years old), including 43 males and 58 females, who were pathologically confirmed to have lung adenocarcinoma, were included in the present study (Table 1). The clinical staging was aligned with the American Joint Committee on Cancer (AJCC) 8th Edition. Among the 101 patients, 44 patients (43.6%) were PD-L1 positive, while 57 patients (56.4%) were PD-L1 negative. All patients were randomly assigned into two groups: the training group included 70 patients (26 PD-L1 positive patients and 44 PD-L1 negative patients), and the validation group included 31 patients (18 PD-L1 positive patients and 13 PD-L1 negative patients).

### 3.2. Radiomic Results

In this study, we catagorized all patient information into three groups: clinical data, thin-slice CT signs and radiomics features (CT), and traditional metabolic parameters and radiomic features from PET (PET). Feature selection and model building are conducted separately using only clinical data, clinical data + CT, and clinical data + CT + PET. The optimal parameters were obtained by training four machine learning models: Tree, Discriminant, Logistic Regression, and Support Vector Machine. The most important features were screened out.

#### 3.2.1. Clinical Data Included in the Model

**Figure 2:**
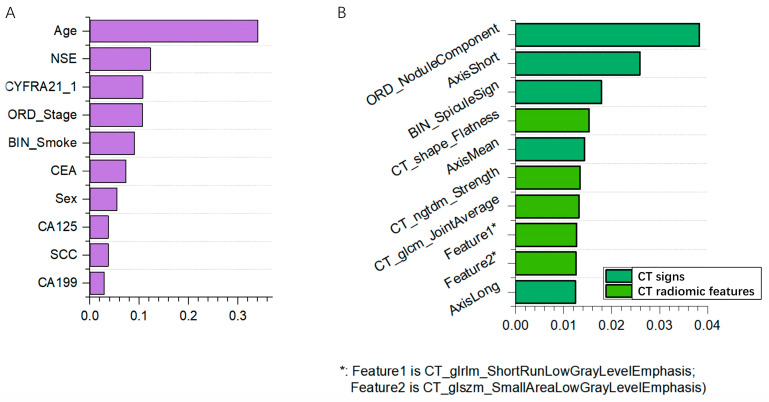
(**A**) Top ten features from clinical data; (**B**) Top ten features from clinical data and CT.

10 out of 11 clinical features were retained after feature denoising with Random Forest. The top ten most important features (Figure 2A) included age, NSE, CYFRA21-1, clinical stage, smoking history, and others.

Feature selection by Stepwise Forward Regression: Clinical stage and gender were selected. In the Tree model, the accuracy of the training group and the validation group was 0.61 and 0.55, respectively. In the Discriminant model, the accuracy of the training group and the validation group was 0.60 and 0.55, respectively. In the Logistic Regression model, the accuracy of the training group and the validation group was 0.66 and 0.55, respectively. In the Support Vector Machine model, the accuracy of the training group and the validation group was 0.67 and 0.55, respectively.

Feature selection by LASSO: Clinical stage, smoking history, and gender were selected. In the Tree model, the accuracy of the training group and the validation group were 0.64 and 0.68, respectively. In the Discriminant model, the accuracy of the training group and the validation group was 0.69 and 0.68, respectively. In the Logistic Regression model, the accuracy of the training group and the validation group was 0.67 and 0.68, respectively. In the Support Vector Machine model, the accuracy of the training group and the validation group was 0.64 and 0.64, respectively.

When only clinical data were included, the accuracy and AUC of Stepwise Forward Regression and LASSO feature selection methods in different models were summarized in Table 2.

#### 3.2.2. Clinical Data and CT Included in the Models

Fifty out of 127 clinical features were selected by feature denoising with Random Forest. The top ten important features (Figure 2B) include five CT signs (nodule component, short axis, spicule sign, etc.) and five CT radiomic features (such as CT shape flatness, CT NGTDM Strength, CT GLCM joint average)

Feature selection by Stepwise Forward Regression: Nodule component, spicule sign, and CT shape flatness were selected. In the Tree model, the accuracy of the training group and the validation group was 0.60 and 0.77, respectively. In the Discriminant model, the accuracy of the training group and the validation group was 0.79 and 0.71, respectively. In the Logistic Regression model, the accuracy of the training group and the validation group was 0.79 and 0.71, respectively. In the Support Vector Machine model, the accuracy of the training group and the validation group was 0.76 and 0.74, respectively.

Feature selection by LASSO: Nodule component and pleural indentation sign were selected. In the Tree model, the accuracy of the training group and the validation group was 0.71 and 0.68, respectively. In the Discriminant model, the accuracy of the training group and the validation group was 0.71 and 0.68, respectively. In the Logistic Regression model, the accuracy of the training group and the validation group was 0.76 and 0.68, respectively. In the Support Vector Machine model, the accuracy of the training group and the validation group was 0.74 and 0.71, respectively.

When clinical data and CT were included, the accuracy and AUC of the Stepwise Forward Regression and LASSO feature selection methods in different models were summarized in Table 3.

#### 3.2.3. Clinical Data and PET/CT Included in the Models

**Figure 3:**
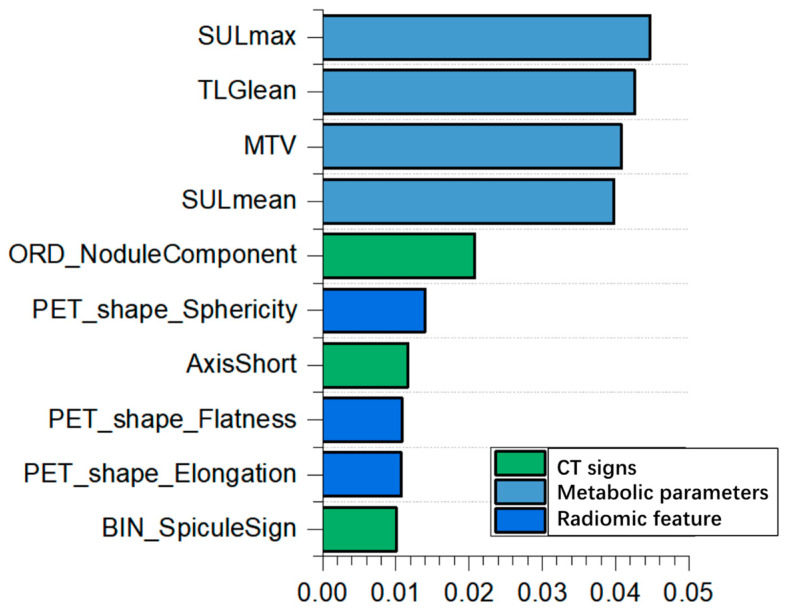
Top ten features of clinical data and PET/CT.

Fifty out of 241 clinical features were selected by feature denoising with Random Forest. The top ten important features (Figure 3) included four traditional metabolic parameters (SULmax, TLGlean, MTV, and SULmean), three CT signs (nodule component, short axis, and spicule sign), and three PET radiomic features (PET Shape Sphericity, PET Shape Flatness, and PET Shape Elongation).

Feature selection by Stepwise Forward Regression: TLGlean, MTV, nodule component, and PET Shape Sphericity were selected. In the Tree model, the accuracy of the training group and the validation group was 0.76 and 0.77, respectively. In the Discriminant model, the accuracy of the training group and the validation group was 0.80 and 0.68, respectively. In the Logistic Regression model, the accuracy of the training group and the validation group was 0.77 and 0.68, respectively. In the Support Vector Machine model, the accuracy of the training group and the validation group was 0.80 and 0.74, respectively.

Feature selection by LASSO: SULmax, MTV, nodule component, PET Shape Sphericity, and spicule sign were selected. In the Tree model, the accuracy of the training group and the validation group was 0.77 and 0.81, respectively. In the Discriminant model, the accuracy of the training group and the validation group was 0.83 and 0.77, respectively. In the Logistic Regression model, the accuracy of the training group and the validation group was 0.81 and 0.74, respectively. In the Support Vector Machine model, the accuracy of the training group and the validation group was 0.81 and 0.77, respectively.

When clinical data and PET/CT were included, the accuracy and AUC of the Stepwise Forward Regression and LASSO feature selection methods in different models were summarized in Table 4.

**Figure 4:**
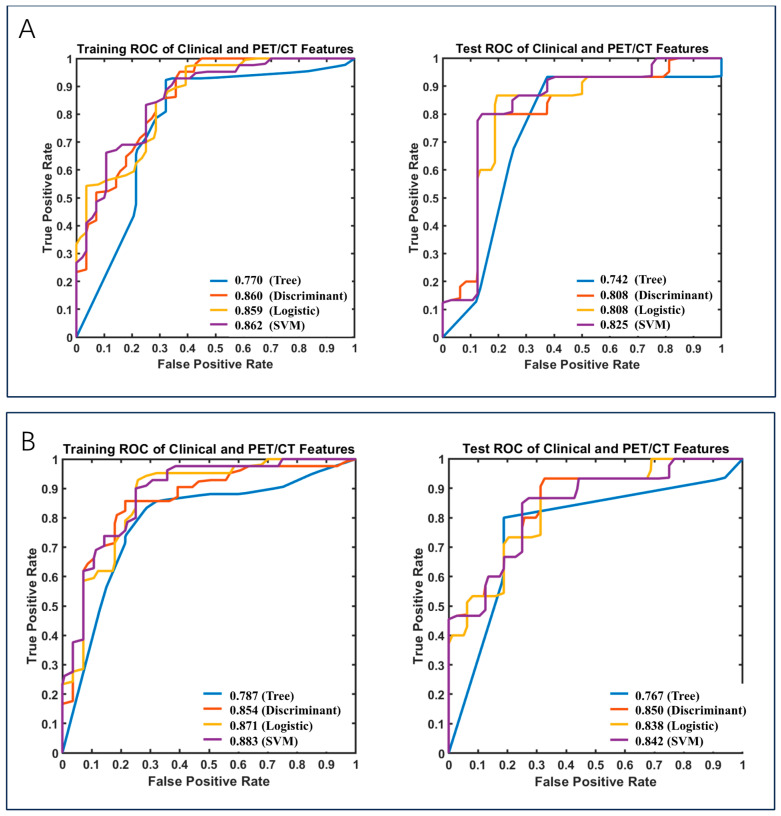
(**A**) The ROC and AUC of the Stepwise Forward Regression selection method; (**B**) The ROC and AUC of the LASSO selection method.

When clinical data and PET/CT were included, the ROC and AUC of the Stepwise Forward Regression and LASSO feature selection methods in different models were shown in Figure 4A,B.

### 3.3. Typical Case

**Figure 5:**
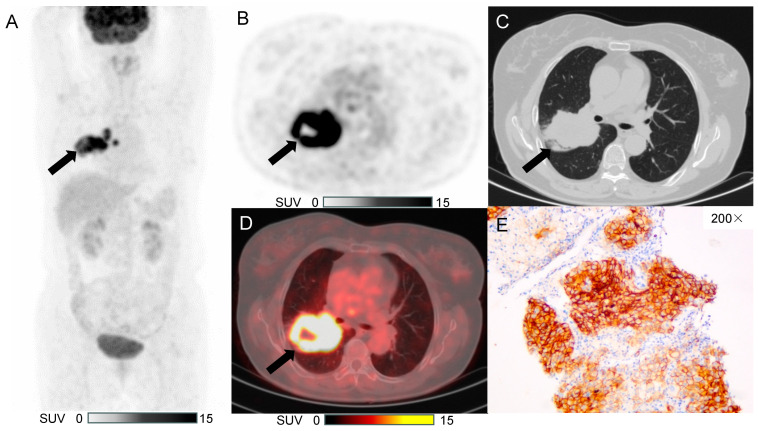
A 64-year-old female, without a smoking history, with a soft tissue nodule in the upper lobe of the right lung, diagnosed with invasive cancer. The arrows indicate the lesions. (**A**) Maximum intensity projection of ^18^F-FDG PET imaging; (**B**) Axial PET scan: SULmax 9.29, SULmean 4.66, MTV 80832, TLGlean 376677.12; (**C**) Axial thin-slice CT, showing that the lesion in the upper lobe of the right lung was solid, with a spicule sign (+), pleural indentation sign (+), and a short axis of 4.4cm. (**D**) Fusion images; (**E**) Immunohistochemical pathology image showing the positive expression (expression rate of 90%) of PD-L1.

To demonstrate the application of the proposed model, a typical case was presented as in Figure 5.

## 4. Discussion

The results of our study demonstrated that the CT signs and PET conventional metabolic parameters showed a significant difference between PD-L1 positive and PD-L1 negative patients with lung adenocarcinoma. CT signs, such as nodule component and short axis, along with PET conventional parameters, such as TLGlean and MTV, may provide important additional information to differentiate PD-L1 positive from PD-L1 negative patients with lung adenocarcinoma.

Immunotherapy has revolutionized treatment strategies in lung cancer. Studies have verified that immunotherapy could significantly improve the progression-free survival (PFS) and overall survival (OS) compared to chemotherapy [7,8,23]. PD-L1 antibodies have been approved by the FDA as first-line therapy for metastatic lung cancer and second-line therapy for advanced lung cancer. Not only do late-stage patients benefit from PD-L1 inhibitors, but early-stage patients as well. Ford et al. [24] were the first to study neoadjuvant checkpoint inhibition in early-stage NSCLC. Their findings indicated that patients tolerated neoadjuvant nivolumab well, and treatment did not delay surgery. The treatment induced a major pathological response in 45% of resection specimens. Tina et al. [25] demonstrated that neoadjuvant therapy combining nivolumab with ipilimumab improved major pathologic response and pathologic complete response rates. They also found that radiographic findings positively correlated with pathologic tumor responses. It is well known that tumors exhibit strong phenotypic differences in patients that can be visualized through imaging. Radiomics is a noninvasive quantitative method that objectively assesses tumor phenotype without observer variation, except for the region of interest predefined by an operator. By extracting hundreds of quantitative features from a myriad of image sections, radiomics provides a far more comprehensive and nuanced representation of the tumor phenotype than the human eye alone.

Previous studies have also established prediction models based on CT images to detect PD-L1 expression levels in lung adenocarcinoma. Sun et al. [20] demonstrated that the predictive model achieved optimal performance when integrating CT radiomics with clinicopathological characteristics, showing superior discriminative ability in both training and validation cohorts. Specifically, the model exhibited an AUC of 0.829, with sensitivity and specificity values of 80.8% and 77.0%, respectively, in the training cohort. These metrics were further validated in an independent cohort, demonstrating comparable performance with an AUC of 0.848, sensitivity of 83.3%, and specificity of 72.4%. Yoon et al. [21] also demonstrated the value of combining CT radiomics and clinicopathologic characteristics for predicting PD-L1 expression, finding that four textural features were related to PD-L1. Although these two studies established satisfactory prediction models of PD-L1 based on CT radiomics, PET information was not discussed. To the best of our knowledge, a prediction model for PD-L1 expression in lung cancer based on PET/CT radiomic features has not been well established. Jiang et al. [26] constructed a prediction model based on PET/CT radiomic features, and their results showed that the model performed better based on CT radiomics than PET/CT radiomics. However, this is not consistent with our results. This discrepancy is likely because their model did not take metabolic parameters into consideration, whereas we measured multiple lean body metabolic parameters, which helped overcome the effects of patient weight. Our study provides evidence that PET radiomics and conventional metabolic parameters may have synergetic effects with CT radiomics in predicting PD-L1 expression. In our results, accuracy and AUC were highest when incorporating clinical characteristics; CT, and PET, and the top four important features were metabolic parameters provided by PET. This may be due to metabolic parameters including more personalized information (e.g., weight, FDG dose, etc.). Takada et al. [18] demonstrated that tumors with higher PD-L1 levels tend to have higher glycolytic metabolism, and SUVmax is a predictor of PD-L1 protein expression in NSCLC patients. Wu et al. [19] verified that PD-L1 is associated with SUVmax and TLG, and SUVmax is an independent predictor of PD-L1 positivity. Cui et al. [27] reached the same conclusion. Lopci et al. [28] found a statistically significant correlation between SUVmax and SUVmean with PD-1 expression. Additionally, we observed that when CT and PET/CT features were incorporated, no clinical variables were selected as the top ten features through the feature selection process. This finding underscores the superior capacity of imaging modalities to provide comprehensive pathological phenotype information compared to clinical data.

Our study has several limitations. First, as a retrospective study, patients were enrolled from a single center, and the sample size was small. We plan to conduct multicenter prospective studies in the future. Second, we used manual segmentation, which is time-consuming and non-reproducible. This was done to ensure accuracy and robustness of the prediction model. Third, the pathological subtype was so that all patients included in this study had adenocarcinoma. Therefore, a variety of pathological subtypes are warranted for the further study. Fourth, this study did not consider the impact of respiratory motion on PET imaging. In the future, combining AI and multimodal imaging could help establish a standardized motion robustness analysis framework to promote reliable application of imaging in precision medicine. Lastly, an independent external test cohort with a larger sample size is needed to further validate our results.

## 5. Conclusions

In the model using PET/CT imaging to predict PD-L1 expression levels in lung adenocarcinoma patients, CT signs—which include the diagnostic experience of imaging physicians—and traditional metabolic parameters of PET play an important role.

## Figures and Tables

**Table 1:** Clinicopathological data of 101 cases.

Clinicopathological Data	Number	Training Group (*n* = 70)		Validation Group (*n* = 31)	
		PD-L1 (+)	PD-L1 (−)	PD-L1 (+)	PD-L1 (−)
Total	101	26	44	18	13
Age					
≥60	51	15	22	10	4
<60	50	11	22	8	9
Gender					
Male	43	12	12	13	6
Female	58	14	32	5	7
Smoking history					
Yes	27	7	7	12	1
No	74	19	37	6	12
Tumor long axis					
>2cm	38	13	10	10	5
≤2cm	63	13	34	8	8
Clinical stage					
I	93	24	43	13	13
II	3	1	1	1	0
III	4	1	0	3	0
IV	1	0	0	1	0
Specimen type					
Biopsy	4	2	1	1	0
Surgery	97	24	43	17	13

**Table 2:** Accuracy and AUC of different models using clinical data only.

	Stepwise Forward Regression				LASSO			
	Training Group		Validation Group		Training Group		Validation Group	
	Accuracy	AUC	Accuracy	AUC	Accuracy	AUC	Accuracy	AUC
Tree	0.61	0.62	0.55	0.55	0.64	0.60	0.68	0.62
Discriminant	0.60	0.67	0.55	0.63	0.69	0.70	0.68	0.65
Logistic Regression	0.66	0.71	0.55	0.63	0.67	0.72	0.68	0.66
SVM	0.67	0.70	0.55	0.63	0.64	0.70	0.65	0.69

**Table 3:** The accuracy and AUC in different models with clinical data and CT.

	Stepwise Forward Regression				LASSO			
	Training Group		Validation Group		Training Group		Validation Group	
	Accuracy	AUC	Accuracy	AUC	Accuracy	AUC	Accuracy	AUC
Tree	0.60	0.58	0.77	0.78	0.71	0.67	0.68	0.71
Discriminant	0.79	0.81	0.71	0.83	0.71	0.77	0.68	0.78
Logistic Regression	0.79	0.80	0.71	0.81	0.76	0.79	0.68	0.78
SVM	0.76	0.80	0.74	0.83	0.74	0.78	0.71	0.79

**Table 4:** The accuracy and AUC in different models with clinical data and PET/CT.

	Stepwise Forward Regression				LASSO			
	Training Group		Validation Group		Training Group		Validation Group	
	Accuracy	AUC	Accuracy	AUC	Accuracy	AUC	Accuracy	AUC
Tree	0.76	0.77	0.77	0.74	0.77	0.79	0.81	0.77
Discriminant	0.80	0.86	0.68	0.81	0.83	0.85	0.77	0.85
Logistic Regression	0.77	0.86	0.68	0.81	0.81	0.87	0.74	0.84
SVM	0.80	0.86	0.74	0.83	0.81	0.88	0.77	0.84

## Data Availability

The raw data supporting the conclusions of this article will be made available by the authors on request.
